# Requirements for the Degree in Medicine

**Published:** 1898-10

**Authors:** 


### Requirements for the Degree in Medicine

AS ADOPTED BY THE AMERICAN MEDICAL ASSOCIATION.

At the recent meeting of the Association the following was unanimously adopted:

"Whereas, The American Medical Association did, at Detroit in 1892, unanimously resolve to demand of all the medical colleges of the United States the adoption and observance of a standard of requirements of all candidates for the degree of doctor of medicine which should in no manner fall below the minimum standard of the Association of American Medical Colleges; and

"Whereas, This demand was sent officially by the permanent secretary to the dean of every medical college in the United States and to every medical journal in the United States, now, therefore, the American Medical Association gives notice that hereafter no professor or other teacher in, nor any graduate of any medical college in the United States, which shall after January 1, 1899, confer the degree of doctor of medicine or receive such degree on any conditions below the published standard of the Association of American Medical Colleges, be allowed to register as either delegate or permanent member of this Association."

"*Resolved*, That the permanent secretary shall within thirty days after this meeting send a certified copy of these resolutions to the dean of each medical college in the United States and to each medical journal in the United States."

